# Fluoroquinolone resistance in urinary tract infections: Epidemiology, mechanisms of action and management strategies

**DOI:** 10.1002/bco2.286

**Published:** 2023-08-31

**Authors:** Daryl Thompson, Jennifer Xu, Joseph Ischia, Damien Bolton

**Affiliations:** ^1^ Department of Surgery The University of Melbourne, Austin Health Heidelberg Victoria Australia; ^2^ Olivia Newton‐John Cancer Research Institute Austin Health Melbourne Victoria Australia

**Keywords:** acute pyelonephritis, chronic prostatitis, fluoroquinolone resistance, multidrug resistance

## Abstract

**Background:**

Fluoroquinolone resistance is an issue of concern amongst physicians worldwide. In urology, fluoroquinolones are often used in the treatment of acute pyelonephritis and prostatitis, as well as infections caused by multidrug‐resistant pathogens.

**Aims:**

We aim to highlight the importance of antimicrobial stewardship and the need for ongoing biomedical research to discover novel agents in our losing battle against resistant pathogens.

**Materials and methods:**

In this review, we survey the literature and summarise fluoroquinolone resistance as it pertains to pyelonephritis and prostatitis, as well as alternative treatment strategies and prevention of multidrug resistance.

**Results:**

The rise of fluoroquinolone resistance in bacteria has reduced the available treatment options, often necessitating hospital admission for intravenous antibiotics, which places an additional burden on both patients and the healthcare system. Many countries such as Australia have attempted to limit fluoroquinolone resistance by imposing strict prescribing criteria, though these efforts have not been entirely successful. Solutions to overcome resistance include prevention, combination therapy and the development of novel antimicrobial agents.

**Conclusions:**

Prevention of the proliferation of resistant organisms by antimicrobial stewardship is paramount, and urologists are obliged to be aware of responsible prescribing practices such as referring to local guidelines when prescribing. By reserving fluoroquinolones for infections in which they are truly indicated and by prescribing based on both patient and local environmental factors, we can preserve this effective resource for future use.

## 1 INTRODUCTION

Fluoroquinolones, an antibiotic class commonly used in urology, are frequently indicated in acute pyelonephritis and bacterial prostatitis. They may also be used in urinary tract infections caused by multidrug‐resistant organisms, or when first‐line antibiotic therapy has failed. Antibiotic resistance, considered a global healthcare crisis by the World Health Organisation, is increasingly becoming a concern regarding these agents.^1^ The objective of this review is to summarise the mechanism, epidemiology, clinical implications and management strategies of fluoroquinolone‐resistant infections. We aim to highlight the importance of antimicrobial stewardship and the need for ongoing biomedical research to discover novel agents in our losing battle against resistant pathogens.

## 2 HISTORY OF QUINOLONE ANTIBIOTICS

The first synthetic quinolone, nalidixic acid, had been used to treat urinary tract infections since the 1960s, though it had relatively low potency. The 1979 discovery of norfloxacin, followed closely by the 1983 patent of ciprofloxacin, propelled fluoroquinolones into widespread use as these newer generation agents were much more clinically effective.^2^ Fluoroquinolones act by interfering with bacterial DNA replication via inhibition of DNA topoisomerase and DNA gyrase, enzymes that are important in bacterial cell division.^2^ They are particularly effective against gram‐negative bacilli, such as *Escherichia coli*, though they also exhibit some activity against gram‐positive bacteria.^2^

Fluoroquinolones demonstrate favourable pharmacokinetics resulting in high concentrations in urine, prostatic tissue and fluid, making them ideal for complex urinary tract infections. They also possess excellent bioavailability following oral administration.^2^ Importantly, they have a notable role as amongst the few oral antibiotics effective against *Pseudomonas aeruginosa*, which is particularly relevant in patients with catheter and urinary calculi‐associated infections.^2^

## 3 FLUOROQUINOLONE RESISTANCE

### 3.1 Mechanism of resistance

With the use of norfloxacin and ciprofloxacin becoming more widespread in the 1980s, so too did the emergence of fluoroquinolone resistance in previously sensitive organisms. Reports on this topic were published as early as 1987, only 4 years after ciprofloxacin was patented.^3^ Further research has shown that resistance can develop after even a single 3‐day course of ciprofloxacin.^4^ The exact mechanism by which organisms develop resistance is often unique to each species. Fluoroquinolone‐resistant members of Enterobacteriaceae*,* the family which includes *E. coli*, *Klebsiella* and *Enterobacter*, have demonstrated mutations in gyrA, a DNA gyrase gene often targeted by antibacterial agents.^5^ Mutations in a gene affecting cell membrane permeability, marA, have also been found in *E. coli*, which is theorised to promote outflux while inhibiting influx of antibiotics through the cell membrane.^5^ *Pseudomonas* species also gain resistance to fluoroquinolones via these mechanisms. *P. aeruginosa* contains 12 different efflux pumps, which work to pump antibiotics out of the cell, and two of these pumps in particular are significantly overexpressed in resistant strains.^6^ More recent research has shown that in addition to de novo genetic mutations, drug resistance can also be transferred from one bacterium to another by way of plasmid‐mediated resistance.^7^

The presence of a drug‐resistant strain in an individual patient may arise from several mechanisms. A patient may become infected with a strain that is already drug resistant, or alternatively, a strain that was sensitive to fluoroquinolones initially may mutate during a single infection and become resistant after treatment, if only transiently. Horcajada et al. demonstrated this in a 2002 study of patients with prostatitis, the majority of whom were initially colonised with ciprofloxacin‐sensitive *E. coli*. After 1 month of fluoroquinolone treatment, half of these patients had been colonised with resistant strains of *E. coli*, which were molecularly distinct from the original sensitive strains and contained at least one mutation, showing how quickly de novo resistance can develop.^8^

### 3.2 Epidemiology

The global burden of drug‐resistant infections is enormous, both in terms of patient mortality and financial costs. Globally, more than 700 000 people die each year because of drug‐resistant infections, with an estimated 23 000 of those occurring in the United States. The Centres for Disease Control and Prevention estimate the cost of antimicrobial resistance in the United States to be $55 billion each year, of which $20 billion is attributed to healthcare costs and $35 billion to loss of productivity.^1^ Although Australia has enjoyed relatively low fluoroquinolone resistance rates in comparison with other developed nations, likely because of strict regulation of its use in both humans and animals, resistance is a topic of increasing concern.^9^

Rates of bacterial resistance to fluoroquinolones vary significantly worldwide and even in a single region can change dramatically over time. For example, *E. coli* strains implicated in acute pyelonephritis in the United States were resistant to ciprofloxacin in 0.2% of patients in 1997, increasing to 1.5% in 2001.^10^ By the early 2010s, resistant *E. coli* was implicated in 6.3% of uncomplicated pyelonephritis and 19.9% of complicated pyelonephritis.^11^ Spain also documented increasing rates of quinolone resistance in community‐acquired infections, quoting 9% in 1992 compared with 17% in 1996.^12^ South Korean patients hospitalised with acute pyelonephritis between 2002 and 2004 cultured quinolone‐resistant *E. coli* at a rate of 19.8%,^13^ and rates were as high as 51.2% in China from 2010 to 2011.^14^ Multidrug‐resistant *Pseudomonas*, a significant issue given the lack of effective treatment options, has also been reported at varying rates worldwide. A 2012 review cited fluoroquinolone‐resistance rates in *Pseudomonas* ranging from 0.6% to 32% depending on location, and also that 27%–72% of patients who were initially colonised with sensitive strains then developed resistant strains at the end of antibiotic therapy.^15^

Resistance rates have been noted to be high in routine testing of relevant patient cohorts, notably in patients undergoing transrectal ultrasound (TRUS) biopsy of prostate. Patients undergoing TRUS biopsy in the United States were colonised with fluoroquinolone‐resistant *E. coli* at a rate of 24% compared with only 15% in controls.^16^ Because TRUS patients are often routinely treated prophylactically with ciprofloxacin, cases of prostatitis post‐biopsy are often due to resistant strains, as found in 85.7% of Egyptian patients.^17^ In prostatitis patients who have not undergone TRUS, resistance to ciprofloxacin has also been shown to be a significant factor. In South Korean patients hospitalised with bacterial prostatitis in 2007, fluoroquinolone‐resistant *E. coli* was found in 23.8% of patients and other resistant pathogens were found in a further 31.6%.^18^

### 3.3 Fluoroquinolone resistance in Australia

In Australia, rates of resistance to fluoroquinolones are relatively low compared with the rest of the world, though they are increasing as in other countries. This can be largely attributed to the lack of fluoroquinolone use in animal agriculture, stringent prescribing regulations and, specifically in Urology, the use of the transperineal approach for prostate biopsies, thus negating the need for prophylactic fluoroquinolone use. Reported rates were 0.4% in 1992, 1% in 1998 and 4.9% in 2006.^9^ The latest data from 2019 suggest that fluoroquinolone resistance in *E. coli* has increased in recent years, particularly in major cities, and is now quoted at a rate of 11%–14%.^19^ *Pseudomonas*, in contrast, remains largely sensitive to ciprofloxacin across Australia with an overall resistance rate of 6.6%.^19^

Contrary to Australia, many European, Asian and North American nations routinely treat livestock with fluoroquinolones, which is thought to confer resistance. When the United States Food and Drug Administration (FDA) approved the use of sarafloxacin treatment for poultry in 1995, fluoroquinolone resistance rates in avian *E. coli* rose from 15% in 1996 to 40% in 1999.^20^ There is also a link between agricultural use of antibiotics and resistance rates in humans. A 1999 study of ciprofloxacin‐resistant *E. coli* in Spain found that resistant strains of *E. coli* in poultry and humans shared more characteristics than did resistant and sensitive strains found in humans only, leading researchers to conclude that strains of ciprofloxacin‐resistant *E. coli* found in humans had likely originated in farm animals.^12^ In Australia, fluoroquinolones are only used sparingly in companion animals and are not used at all in animal agriculture. As a result resistant strains found in animals are most often molecularly distinct from those found in humans, indicating separate origins.^21^

A further reason for the relative scarcity of fluoroquinolone resistance in Australia is the stringency of prescribing regulations. Although international guidelines such as the European Association of Urology (EAU) recommend ciprofloxacin as first‐line therapy for uncomplicated pyelonephritis,^22^ the Australian Therapeutic Guidelines favour amoxicillin and clavulanic acid and only recommend fluoroquinolones in cases of penicillin hypersensitivity or if there is proven resistance to first‐line antibiotics.^23^ Fluoroquinolones are recommended in chronic prostatitis, but not acute, and in any urinary tract infection in which *Pseudomonas* is proven to be the causative organism.^23^ Both ciprofloxacin and norfloxacin are restricted by the Australian Pharmaceutical Benefits Scheme and require an authority code to prescribe.^24^

## 4 CLINICAL IMPLICATIONS OF FLUOROQUINOLONE‐RESISTANT INFECTIONS

### 4.1 Risk factors

Fluoroquinolone‐resistant infections are often associated with known risk factors. Acute pyelonephritis is more likely to be associated with resistant *E. coli* when patients have recently used fluoroquinolones, been hospitalised, or stay in a long‐term care facility.^25^ Pathogens acquired in hospital are more likely to be multidrug‐resistant than those acquired in the community, and indeed healthcare‐associated *E. coli* is more likely to be fluoroquinolone‐resistant.^26^ Patient factors may also contribute to drug resistance, with studies finding that patients who are pregnant, male sex, immunosuppressed, or who have current or pre‐existing functional or anatomical urinary tract abnormalities are more likely to culture fluoroquinolone‐resistant *E. coli* as the causative pathogen in pyelonephritis.^11^ Similarly for acute bacterial prostatitis (ABP), patient factors including advanced age, large prostate size and high post‐void residual volumes^27^ have been identified as independent predictors of the development of fluoroquinolone resistance. Furthermore, routine prophylactic use of fluoroquinolone in patients undergoing TRUS biopsy of the prostate is a key driver of colonisation with resistant *E. coli* ^28^ and increases the incidence of post‐biopsy infectious complications such as prostatitis and septicaemia.^29^

### 4.2 Pathogenesis

Although drug‐resistant pathogens may be more difficult to treat because of limited antibiotic options, several studies have found that resistant *E. coli* may be less virulent than their sensitive counterparts. Vila et al. studied virulence factors in *E. coli* isolates from female patients with acute pyelonephritis and showed that resistant strains had significantly fewer virulence factors than sensitive strains.^30^ This was followed by an in vitro study by Horcajada et al., which reported similar findings and concluded that there was a direct association between quinolone resistance and loss of virulence factors.^31^ However, lower virulence does not necessarily translate into a meaningful difference in terms of clinical course for patients with invasive urinary tract infections such as pyelonephritis and bacterial prostatitis, as other studies have found that bacteraemia rates do not differ significantly between infections caused by resistant versus susceptible *E. coli*.^32^ In contrast, *Pseudomonas* has been reported to exhibit a positive correlation between virulence and quinolone resistance, often resulting in worse clinical outcomes for patients infected with resistant strains.^33^

## 5 MANAGEMENT OF FLUOROQUINOLONE‐RESISTANT INFECTIONS

### 5.1 Prevention

A vital step in the management of drug resistance is prevention, usually by encouraging and enforcing responsible prescribing practices. It was estimated in 2010 that 20%–50% of all antibiotics prescribed in hospitals worldwide are unnecessary.^34^ In a prospective study of 185 urinalyses, investigators demonstrated that only 20% of urinary tract infections in the community were managed according to guideline principles and that fluoroquinolones made up 59.5% of all antibiotics prescribed, thus highlighting an area for improvement with targeted education and regulation. However, recent reports from the Antimicrobial Use and Resistance in Australia (AURA) Surveillance System indicate that despite extensive strategies, this trend has not improved.^19^ In fact, Australia continues to use more antibiotics per capita than other developed nations, including Canada, the United Kingdom and the European Union.^19^

Integration of multidisciplinary antimicrobial stewardship programs and implementation of dual fluoroquinolone formulary have been shown to result in a 30% decrease in empirical fluoroquinolone prescribing and an overall 10% improvement in *P. aeruginosa* susceptibility.^35^ For patients undergoing prostate biopsy who are at increased risk of resistant prostatitis, a transperineal approach is safer with lower rates of infection and equivalent diagnostic accuracy for cancer compared with the transrectal approach,^36^ negating the need for prophylactic fluoroquinolones. Where the transperineal approach is not appropriate, targeted prophylactic antibiotics guided by pre‐operative rectal swabs can reduce post‐biopsy infection and sepsis^37^ in this patient population.

### 5.2 Clinical management

When considering quinolone treatment in cases of culture‐pending pyelonephritis, both environmental and patient variables must be considered. Local resistance rates are an important factor that will dictate prescribing practices. Both the EAU Guidelines and the American Academy of Family Physicians (AAFP) recommend prescribing fluoroquinolones for uncomplicated pyelonephritis only when local resistance rates are below 10%.^22^, ^38^ As of 2019, all areas of Australia, including regional and remote centres, have an estimated *E. coli* resistance rate of above 10%, hence the recommendation for amoxicillin‐clavulanic acid as first‐line therapy for pyelonephritis.^19^ Because recent hospitalisation, particularly on a urology ward, is strongly associated with ciprofloxacin resistance, as is recent fluoroquinolone use, patients with these risk factors should not be prescribed fluoroquinolones.^22^

Fluoroquinolones remain first‐line treatment only in chronic, not acute, cases of bacterial prostatitis because of their favourable pharmacokinetic properties, safety profile and efficacy against common causative pathogens including *P. aeruginosa* and *C. trochomatis*.^39^ In resistant cases of bacterial prostatitis, third‐generation cephalosporins or carbapenems have been shown to be effective^40^ with evidence emerging for the role of fosfomycin in patients with multidrug resistance (MDR) chronic prostatitis.^41^

Despite stringent prescribing regulations, cases of quinolone‐resistant urinary tract infection continue to occur at increasing rates in Australia and present a challenge to urologists. Bacteria that are resistant to fluoroquinolones are likely to be resistant to other antimicrobials. In Australia, *E. coli* cultured from urine is most likely to be resistant to ampicillin/amoxicillin (44.9%), trimethoprim (24%) and fluoroquinolones (11.4%), whereas *E. coli* cultured from blood is often resistant to ampicillin/amoxicillin (54%), trimethoprim‐sulfamethoxazole (28.4%) and cefazolin (27.3%).^19^ However, there is often a viable alternative found on culture sensitivities, though this may be an intravenous preparation necessitating hospital admission. Most bacteraemia‐causing *E. coli* will be sensitive to piperacillin/tazobactam, with a resistance rate of only 6.1%, and essentially, all *E. coli* in Australia is sensitive to meropenem with a resistance rate of 0%.^19^ Gentamicin is also highly effective against both urine‐ and blood‐culture‐positive *E. coli*, with resistance rates of 6% and 8.4%, respectively.^19^ Antibiotic choice in cases of fluoroquinolone‐resistant *E. coli* will therefore depend on extended sensitivities, local policy and patient factors such as renal function.

Multidrug‐resistant *Pseudomonas* strains are much rarer in Australia. Although fluoroquinolone resistance is the most common antimicrobial resistance in *P. aeruginosa* at 6.6%, resistance to other antimicrobials occurs at similar frequencies. Resistance has been reported to piperacillin/tazobactam (5.9%), ceftazidime (4.5%), gentamicin (4.2%) and meropenem (3.1%). The antimicrobial most likely to be effective against resistant *Pseudomonas* is tobramycin, but even this carries a resistance rate of 1.2%.^19^

### 5.3 Emerging evidence for future practice

With the challenge of treating patients with acute pyelonephritis caused by multidrug‐resistant *Pseudomonas*, there is a demand for new treatment regimens or novel antimicrobial agents. Various in vitro studies have reported suppression of MDR with certain combinations, including fosfomycin and amikacin,^42^ imipenem and tobramycin,^43^ and meropenem and ciprofloxacin.^44^ Literature on the development of novel agents is limited, and most new agents are still in their infancy with results from in vitro models only.^45^ Other studied mechanisms of action include targeting immunomodulation with drugs such as clarithromycin. A 2004 study of rabbits with experimental sepsis secondary to multidrug‐resistant *Pseudomonas* pyelonephritis reported that animals treated with clarithromycin experienced prolonged survival compared with untreated rabbits, despite having similar numbers of viable intrarenal bacterial cells.^46^ Although much of this research shows promise, there have not yet been any human clinical trials investigating novel treatments for multidrug‐resistant *Pseudomonas*, making antimicrobial stewardship imperative in prolonging the effectiveness of currently available treatments.

Relative to ABP, treatment of chronic bacterial prostatitis (CBP) is more challenging as few oral antimicrobials can achieve sufficiently effective bactericidal concentrations within prostatic tissue.^47^ This has been postulated to be related to the difficulty of eradicating protected bacterial microcolonies in a chronic infection‐induced microenvironment.^48^ With resistance to fluoroquinolones rising at an alarming rate, options for fluoroquinolone‐resistant CBP are severely limited. Attempts to circumnavigate this have been documented to a limited extent in the literature and include electrode pharmaphoresis via injection of water‐soluble antibiotics to perineal skin^49^ as well as bacteriophage therapy.^50^

## 6 CONCLUSION

Fluoroquinolones are a key class of antibiotics as they have traditionally been very effective against complex urinary tract infections. Increasing rates of resistance to these agents should be a cause for concern. Although novel agents are in development, they are likely still many years from clinical availability. Prevention of the proliferation of resistant organisms by antimicrobial stewardship is paramount, and urologists are obliged to be aware of responsible prescribing practices such as referring to local guidelines when prescribing. By reserving fluoroquinolones for infections in which they are truly indicated, and by prescribing based on both patient and local environmental factors, we can preserve this effective resource for future use.

## AUTHOR CONTRIBUTIONS

Conceptualisation, Damien Bolton. Methodology, Daryl Thompson, Damien Bolton; Software, N/A; Validation, Daryl Thompson, Jennifer Xu; Formal Analysis, N/A; Investigation, Daryl Thompson, Jennifer Xu; Resources, Damien Bolton; Data Curation, Daryl Thompson, Jennifer Xu; Writing—Original Draft Preparation, Daryl Thompson, Jennifer Xu; Writing—Review and Editing, Daryl Thompson, Joseph Ischia; Damien Bolton; Visualisation, Damien Bolton; Supervision, Joseph Ischia, Damien Bolton; Project Administration, Damien Bolton; Funding Acquisition, N/A.

## CONFLICT OF INTEREST STATEMENT

The authors report no conflicts of interest.
